# Development of long-circulating lapachol nanoparticles: formation, characterization, pharmacokinetics, distribution and cytotoxicity

**DOI:** 10.1039/d0ra05752e

**Published:** 2020-08-14

**Authors:** Qunying Chen, Lu Bai, Xuelin Zhou, Pingxiang Xu, Xiaorong Li, Huanli Xu, Yuanyuan Zheng, Yuming Zhao, Shousi Lu, Ming Xue

**Affiliations:** Department of Pharmacology, Beijing Laboratory for Biomedical Detection Technology and Instrument, School of Basic Medical Sciences, Capital Medical University Beijing 100069 China xuem@ccmu.edu.cn; China Rehabilitation Research Center Beijing China luss12@163.com

## Abstract

Lapachol is an active compound for the treatment of malignant brain glioma. However, its physicochemical properties limit its clinical application. The purpose of this study is to develop a nano-drug delivery system (LPC-LP) loaded with lapachol (LPC), which remarkably prolongs the half-life in the body, and increases the brain intake, therefore, achieving a better anticancer effect in the treatment of glioma. In order to optimize the formulation of liposomes, an orthogonal design was adopted with entrapment efficiency (EE) as the index. The characterization of the optimized formulation was evaluated *in vitro*. To assess the safety profile and effect of LPC-LP, a rapid and sensitive ultra-fast liquid chromatography with tandem mass spectrometry (UPLC-MS/MS) method was developed for studying the pharmacokinetics and brain distribution of LPC-LP and LPC. Finally, the cytotoxicity of the two preparations on C6 cells was studied by the MTT assay. The results showed that the average particle size of LPC-LP was 85.92 ± 2.35 nm, the EE of liposomes was 92.52 ± 1.81%, and the charge potential was −40.70 ± 9.20 mV. An *in vitro* release study showed that the release of lapachol from LPC-LP was delayed compared to LPC, indicating that LPC-LP was a sustained and controlled release system. The UPLC-MS/MS method was fully validated in both plasma and brain tissue according to the Food and Drug Administration (FDA) recommended guidelines, and successfully used for quantification of lapachol *in vivo*. After intravenous administration, LPC-LP prolonged circulation time of lapachol in the body and increased brain intake. Besides, the MTT results revealed that the IC_50_ value of LPC-LP on C6 cells significantly decreased, compared with LPC, which further confirmed that LPC-LP enhanced the inhibition of C6 cells and improved the anti-glioma effect. In conclusion, LPC-LP could serve as a promising candidate for the clinical application of lapachol in the treatment of glioma.

## Abstract 2

LPC-LP is a promising and potential nanoparticle in the treatment of glioma.

## Graphical abstract

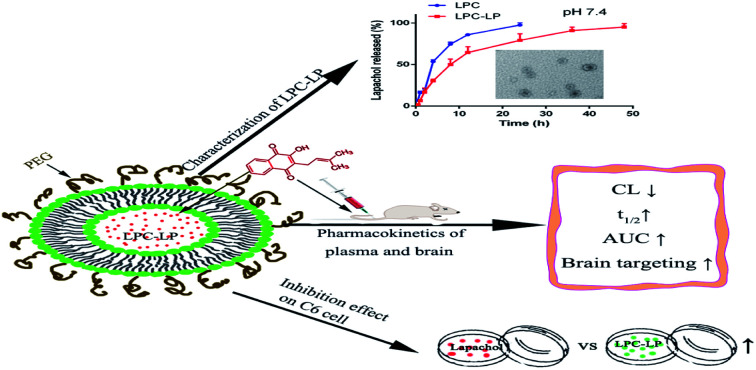

## Introduction

Glioma is an aggressive type of brain cancer with the characteristics of fast growth and penetration.^1^ The incidence of glioma is high, accounting for 40% of all primary brain tumors.^2^ Although there have been significant advances in the treatment of glioma in recent years, the prognosis of patients with glioma is poor.^3,4^ Among many treatments for glioma, chemotherapy is the most common auxiliary treatment for brain glioma.^5^ However, the therapeutic effect is not ideal because of multidrug resistance (MDR). The increase and spread of resistance to the classical anticancer drugs is the main cause of the poor prognosis of this life-threatening disease. Therefore, new drugs need to be developed urgently to achieve better treatment and prolong the life of the patients.

At present, more than 60% of drugs for the treatment of cancers derive from phytochemicals or their derivatives,^6–9^ such as paclitaxel,^10–12^ camptothecin,^13–15^ curcumin,^16–18^ resveratrol,^19–21^*etc.* A large number of studies found that the effective active ingredients extracted from plants not only had anti-tumor activity but also had relatively good safety. Therefore, drugs derived from plants have a broad prospect for the treatment of cancers. Lapachol is a kind of naphthoquinone, obtained from the *Bignoniaceae* family mainly.^22^ The naphtalenic ring of lapachol participated in the redox cycle in cells, and formed conjugated intermediate after reduction and activation, which acted as a powerful alkylating agent. This unique structure was an essential connector of electron transfer in the process of biological metabolism and was also the structural basis of its various biological activities and extensive pharmacological effects, such as anticarcinogenic, anti-inflammatory, antimalarial and antiviral.^23^ Anticancer studies of lapachol were traced to the 1970s. Lapachol was effective against many tumor cells, such as Ehrlich's carcinoma, PC-3 prostate cancer, Lovo colon cancer cell, *etc.*^2,24,25^ In brazil, lapachol was used for adjunctive treatment of cancer in clinic.^26^ Our previous research showed that lapachol inhibited the proliferation of C6 cells with a dose-dependent manner *in vitro*, significantly reduced the tumor volume in the brain of glioma-bearing rat, and prolonged the life of rats without affecting the body-weight *in vivo*, which suggested that lapachol was a promising drug for the malignant glioma therapy.^2^ However, clinical promotion of lapachol was restricted due to drawbacks of lapachol including low aqueous solubility in water, toxic side effects and poor bioavailability. Moreover, in our later studies, we found that the brain distribution of lapachol remained at a very low level, the ratio of the concentration–time curve (AUC) in brain/AUC in plasma was only 0.028%, the rate of elimination was much faster in the brain than that of in the plasma, and the main cause of this phenomenon was the blood–brain–barrier (BBB).^27^

The tight junction of the vascular endothelial cells (ECS) and efflux transporters overexpressed on the surface of BBB are two main factors to protect the brain and maintain homeostasis of the brain parenchymal microenvironment, but they also limit the delivery of therapeutic drugs to the brain.^28,29^ With the development of nanotechnology, many delivery systems are used to overcome the physicochemical limitations of drugs, increase the accumulation of target organs, and control the release speed of drugs in the body, such as liposome, micelle, dendrimer and so on.^30–39^ Among them, liposome has been frequently used as carrier for brain target due to its lower toxicity, higher lipophilicity and better biocompatibility, compared with other nanoparticles.^40^ Polyethylene glycol (PEG) is a kind of hydrophilic polymer which is often modified on the surface of liposomes to reduce interaction between nanoparticles and enzymes *in vivo*, make them invisible to mononuclear phagocyte system (MPS), thus prolong circulation in the plasma, and then further increase the delivery to the brain. Therefore, the efficacy of the drug is improved.^30,31,38^ Besides, some studies showed that PEGylated liposomes increased the delivery of drugs with active efflux properties into the brain.^41,42^ Therefore, the PEGylated liposome is an ideal drug delivery system for the treatment of brain diseases.

The pharmacokinetics study could help to assess the safety margin and profile of drugs with potential clinical applications in nanotechnology. It is an essential step in the preliminary stage for designing and screening drug candidates.^43^ In order to compare the pharmacokinetics and brain distribution between lapachol and its nano-liposome, a validated bioanalytical assay for determination of lapachol is needed. There were only few studies on the pharmacokinetics of lapachol *in vivo*.^27^ In previous research, we had established a HPLC-MS method to detect lapachol *in vivo*, with the lower limit of quantitation (LLOQ) of 0.5 μg ml^−1^, and the method needed almost 10 min for runtime. It took too long time to test a sample, and the LLOQ was high. Besides, after the administration of two formulations, the concentration range of drugs *in vivo* is too wide to use this method.^44^ Therefore, further optimization should be conducted on this method, and the ultra-fast liquid chromatography with tandem mass spectrometry (UFLC-MS/MS) can offer a powerful tool.

Lapachol is a promising drug for the treatment of malignant glioma. Nevertheless, the defects of physicochemical property limit its clinical application. Thereby, the study of lapachol-liposome (LPC-LP) is an essential step in the development of lapachol for the treatment of brain glioma. In our study, PEGylated liposome loaded with lapachol was obtained for the first time. A rapid, sensitive UFLC-MS/MS method was established to detect lapachol with a wide concentration range *in vivo*. It was applied successfully to the study on the pharmacokinetics and brain distribution of LPC-LP. Finally, the anticancer efficacy of LPC-LP was evaluated *in vitro*. Thereby, the objective of this study is to develop a kind of nanoparticle loaded with lapachol, which can optimize the pharmacokinetic characteristics of lapachol *in vivo*, increase the delivery of drugs to the brain, and achieve better anticancer efficacy for the treatment of glioma.

## Material and methods

### Materials

Lapachol [2-hydroxy-3-(3-methyl-2-butenyl)-1,4-naphthoquinone] was purchased from Nanjing Jiancheng Bioengineering Institute (Nanjing, China). DSPE-mPEG2000 [1,2-distearoyl-*sn-glycero*-3-phosphoethanolamine-*N*-(maleimide-2000)] was provided by the Corden Pharma (Liestal, Switzerland). Phosphatidylcholine was obtained from Macklin (Shanghai, China). Cholesterol was purchased from ABCONE (Shanghai, China). Acetonitrile of HPLC grade was supported by Fisher Scientific (Fair Lawn, NJ, USA), thiazolyl blue tetrazolium bromide (MTT) assay kits were purchased from Solarbio (Beijing, China). All other solvent and chemicals were of analytical grade.

### Preparation of LPC-LP

**Scheme 1:**
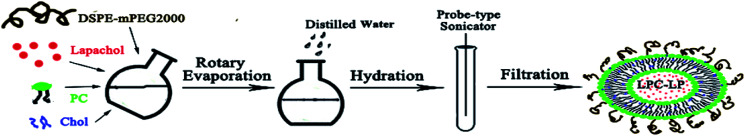
Schematic illustration of the preparation of LPC-CP.

LPC-LP was prepared by the thin-film rotary evaporation method (Scheme 1).^38^ Briefly, lapachol, phosphatidylcholine (PC), cholesterol (Chol) and DSPE-mPEG2000 were dissolved in dichloromethane respectively, and mixed in the round-bottom flask. Subsequently, the organic solvent was removed by vacuum rotary evaporation to prepare lipid film, with the temperature at 40 °C and rotate speed of 120 rpm min^−1^. After the thin film was hydrated by normal saline, the suspension was transferred to the test tube and treated with a probe-type sonicator (240 W) to make the solution translucent. Finally, the solution was extruded through 0.22 μm polycarbonate filters to obtain the LPC-LP.

### Optimization of LPC-LP formulation

In order to obtain an optimal formulation of LPC-LP, the orthogonal test was employed with four-factor and three-level. The four factors included PC : Chol mole ratio (factor A), PC : DSPE-mPEG2000 mole ratio (factor B), PC : LPC mole ratio (factor C) and the ultrasonic time (factor D).^7,39,45^ Each factor was studied for three levels, and the encapsulation efficiency (EE) was selected as the evaluation indicator of the formulations to screen out the optimal formulation, as showed in Table 1.

**Table 1:** Factors and levels of orthogonal experimental design

Levels	Factors			
	A	B	C	D
	PC : Chol (n/n)	PC : DSPE-mPEG2000 (n/n)	PC : LPC (n/n)	Ultrasonic time (min)
1	8 : 1	34 : 1	100 : 1	5
2	16 : 1	68 : 1	50 : 1	10
3	32 : 1	102 : 1	20 : 1	15

### Characterization of LPC-LP

Before the measurement, the freshly made nanoparticles were diluted with distilled water. The mean particle size, particle dispersion index (PDI) and zeta potential (ZP) of LPC-LP were determined by the Zetasizer Nano S90 (Malvern Instruments, U.K.). All the measurements were performed in triplicate. For the morphology of LPC-LP, the diluted solution was dropped onto the film-coated copper grid. When the solution was dry, the samples were observed by the JEM-2010 electron microscope (Tokyo, Japan) with the accelerating voltage of 80 kV.

The concentration of lapachol was detected by the Agilent 1100 high-performance liquid chromatography (HPLC) system (Agilent Corporation, USA), the separation was performed on the ZORBAX Eclipse plus-C18 column (3.5 μm, 100 × 2.1 mm; Agilent Corporation) with a flow rate of 1.0 ml min^−1^. The mobile phase was acetonitrile/0.1% formic acid water (45 : 55, v/v), and the detection wavelength of lapachol was at 249 nm. The amount of lapachol encapsulated in LPC-LP was achieved by following steps: firstly, LPC-LP was dissolved in methanol. Subsequently, the solution was sonicated for 30 min and centrifuged at 13 000 rpm for 10 min. After filtration through 0.22 μm membranes, 15 μl of solution was injected into the HPLC, and the amount of lapachol in LPC-LP was finally measured. EE of LPC was calculated as the following formula:EE (%) = *W*_loaded_/*W*_total_ × 100%where the *W*_loaded_ was the amount of lapachol encapsulated in LPC-LP, the *W*_total_ was the total amount of lapachol added.

The *in vitro* release of lapachol from LPC-LP and LPC was carried out by the dialysis bag diffusion technique.^46,47^ Briefly, LPC-LP and LPC (0.2 mg ml^−1^, 5 ml) were placed in the dialysis bags (MWCO: 500) which were soaked in PBS for 24 h prior to the experimentation, then these bags with both ends fastened were put in the beakers filled with 50 ml of PBS (pH 7.4) containing 1% SDS, which acted as the release medium. The temperature of the water bath was kept at 37 °C, and the stirring speed was 100 rpm min^−1^. After incubation for 0.5, 1, 2, 4, 6, 8, 12, 24, 36 and 48 h, 0.2 ml of dissolution medium was collected at each predetermined time point respectively, and fresh medium with the same volume and temperature was supplied simultaneously to maintain sink conditions. The concentration of lapachol in release medium was detected by the HPLC system as described above. All the release experiments were performed in triplicate, and the results were expressed as mean ± standard deviation.

### LC-MS/MS determination for LPC-LP and LPC *in vivo*

The UPLC-MS/MS system consisted of a UPLC system (Agilent Technologies, Palo Alto, CA, USA) including a G1316C vacuum degasser, a G4220A binary pump, G4226A auto-sampler, and a triple quadrupole mass spectrometer equipped with electrospray ionization (AB 6500 Triple Quad, AB SCIX, USA). The separation was achieved on a ZORBAX SB-C18 (100 mm × 2.1 mm i.d., 1.8 μm, Agilent Technologies, USA) coupled with a C18 guard column (5 mm × 2.1 mm i.d., 5 μm, Agilent Technologies, USA). The gradient elution of 0.02% formic acid aqueous solution and acetonitrile changed linearly from 40 : 60 (v/v) to 15 : 85 (v/v) at a flow rate of 0.3 ml min^−1^. The running time per sample was approximately 5 min. The mass spectrometric measurement was performed in the negative ion mode, with curtain gas at 3.8 l min^−1^, ion spray voltage of 4.5 kV, and temperature of 550 °C. The optimized precursor-to-product ion transitions with the multiple reactions monitoring mode (MRM) were the *m*/*z* 241 → 186 for lapachol, and the *m*/*z* 255 → 119 for isoliquiritigenin (IS).

The plasma samples and brain homogenates were treated with protein precipitation, 50 μl of plasma sample was spiked with 50 μl of acetonitrile and IS (5 μg ml^−1^). The mixture was mixed by vortexing for 1 min, and then centrifuged at 13 000 rpm for 10 min at 4 °C. The supernatant was filtered through nylon filters (0.22 μm), and 5 μl aliquot was analyzed by the UPLC-MS/MS system.

Standard stock solutions of lapachol (1 mg ml^−1^) and isoliquiritigenin (IS, 1 mg ml^−1^) were prepared in acetonitrile. The calibration samples for low and high concentrations range of lapachol (low level: 1000, 200, 100, 20, 10 and 5 ng ml^−1^ for analysis of plasma and brain tissue; high level: 100, 50, 20, 5, 2 and 1 μg ml^−1^ for analysis of plasma) were obtained by diluting the stock solution with acetonitrile. The standard solution of isoliquiritigenin (IS) was also diluted to the concentration of 5 μg ml^−1^ with acetonitrile. All the solutions were kept at 20 °C until analysis. The quality control (QC) samples (5, 100 and 1000 ng ml^−1^ for plasma and brain tissue analysis; 1, 20 and 100 μg ml^−1^ for plasma analysis) were prepared in the same way as described above.

The method validation was carried out according to the Food and Drug Administration (FDA) guidelines on linearity, selectivity, accuracy, precision, recovery, stability and matrix effect in two biological matrices: plasma and brain tissue.

### Pharmacokinetics and brain distribution of LPC-LP and LPC

Adult male Sprague–Dawley rats (260 ± 30 g) obtained from the Laboratory Animals Center of Capital Medical University (LAC, CMU, Beijing, China) were used to analyze the pharmacokinetic profile of lapachol *in vivo*. All animal procedures were performed in accordance with the Guidelines for Care and Use of Laboratory Animals of Capital Medical University and approved by the Animal Ethics Committee of Capital Medical University (approval ID: AEEI-2017-131). The rats were randomly divided into two groups, with six rats in each group and fasted overnight before the experiment. LPC (dissolved in normal saline coupled with 40% propanediol) and LPC-LP were injected *via* the tail vein at a dosage of 6.8 mg kg^−1^. The blood samples (300 μl) were obtained from the jugular vein and collected in the heparin pre-treated polypropylene centrifuge tubes before administration and post-dosing at 0.03, 0.08, 0.17, 0.33, 0.5, 1, 2, 4, 8, 12 and 24 h, respectively. The plasma was isolated by centrifuging blood samples at 13 000 rpm for 10 min at 4 °C, and then stored at −80 °C for further analysis. The pharmacokinetic parameters of lapachol from two formulations were calculated by the ware DAS Version 2.0 (Chinese Pharmacological Society, Beijing, China).

In order to verify the brain-targeting, brain distribution of lapachol was performed in ICR mice (20 ± 3 g), which also were obtained from the Laboratory Animals Center of Capital Medical University (LAC, CMU, Beijing, China), the mice were divided into two groups and injected two formulations at a dose of 9.5 mg kg^−1^. At each predetermined time point (0.17, 1, 4 and 8 h) after dosing, three mice were sacrificed and dissected to collect the brain. The brain tissues were weighed and homogenized with cold saline (w/v = 1 : 2). Subsequently, the brain homogenates were centrifuged at 13 000 rpm for 10 min. The supernatant was transferred into the tube, and stored at −80 °C until analysis. The maximum concentration (*C*_max_) in brain was obtained from observed data, and AUC was calculated by the linear trapezoidal method.^48^

### Cytotoxic effect of LPC-LP and LPC on C6 cells

To compare the proliferation inhibition effect of LPC-LP and LPC, C6 cells were passaged, digested, and centrifuged to prepare cell suspension, then the cell density was adjusted to 4 × 10^3^ cells per ml with Dulbecco's modified eagle medium (DMEM), 100 μl per well was seeded in 96-well plates and cultured under an atmosphere of 5% CO_2_ at 37 °C. Meanwhile, LPC and LPC-LP were diluted with DMEM medium to the desired concentration gradients: 20, 10, 5, 2, 1 and 0.5 μM. After incubation for 24 h, the upper-medium of cells was discarded and replaced with the DMEM medium containing different concentrations of LPC-LP and LPC, three replicate wells were set for each concentration, and the control groups were treated with fresh DMEM.^5^ The culture continued for another 48 h after administration, and the cellular proliferation inhibition rate of LPC-LP and LPC was determined by MTT assay. Briefly, after washing the cells with PBS for three times, the MTT solution was added to the wells and incubated for another 2 h, then the optical density (OD) was detected by spectrophotometer at a wavelength of 570 nm.^49^ The inhibitory rate (IR) was calculated by the following formula:IR% = 1 − (OD for the treated cells/OD for the control cells) × 100%

### Statistical analysis

The results of the experiments were presented as mean ± standard deviation (SD). Statistics were analyzed by the SPSS 19.0 statistical software, and differences between groups were determined with one-way ANOVA method. The value of P less than 0.05 was considered a significant difference.

## Results and discussion

### Preparation and optimization of the LPC-LP formulation

As drug carriers, liposomes are considered to be the most mature and promising nano-drug delivery system with good morphological fluidity, biocompatibility and non-toxicity. Especially in the treatment of tumor, it can increase the affinity with tumor cells, enhance the uptake of drugs by tumor cells, overcome the drug resistance, reduce the dosage, improve the efficacy, and reduce the side effects.^7,50,51^ When the surface of the liposome is modified with PEG, long-circulating liposomes are formed, which can effectively prolong the circulation time of the drug in the body, improve the bioavailability, thus further improve the therapeutic effect. In this study, PEG-liposomes for lapachol were prepared by the thin-film rotary evaporation method for the first time, and the preparation process was optimized. For optimization of the LPC-LP formulation, EE as an index of evaluation was detected by HPLC. Lapachol was extremely difficult to dissolve in water, and the concentration of lapachol in saturated solution could not be detected by HPLC. Therefore, EE of LPC-LP was determined by direct filtration instead of over-speed separation of LPC-LP in this study. According to the orthogonal test as L9 (3^4^), nine of the formulations were designed, the results were shown in Table 2. The order of extremum values (*R*) was C > A > D > B, indicating that the ratio of PC : LPC had the greatest influence on the EE of LPC-LP, followed by the ratio of PC : Chol, and then the ultrasonic time. The ratio of PC–DSPE-mPEG2000 had the least effect, which was consistent with the results of the analysis of variance. The influence order of factors at different levels on the EE of LPC-LP was displayed as follows: A2 > A3 > A1; B1 > B3 > B2; C1 > C2 > C3; D3 > D2 > D1. In summary, the optimal synthetic condition of LPC-LP was A2B1C1D3, *i.e.*, the molar ratio of PC–Chol was 16 : 1, PC : DSPE-mPEG2000 was 34 : 1, PC : LPC was 100 : 1, and the ultrasound time was 15 min. Three batches of LPC-LP were prepared according to the above optimum process, and the EE was 92.52 ± 1.81%.

**Table 2:** L9 (3^4^) orthogonal experimental design and the results of LPC-LP preparations^a^

No.	Factors				EE%
	A	B	C	D	
1	8 : 1	34 : 1	100 : 1	5	58.51
2	8 : 1	68 : 1	50 : 1	10	29.00
3	8 : 1	102 : 1	20 : 1	15	13.77
4	16 : 1	34 : 1	50 : 1	15	61.84
5	16 : 1	68 : 1	20 : 1	5	21.15
6	16 : 1	102 : 1	100 : 1	10	83.74
7	32 : 1	34 : 1	20 : 1	10	21.88
8	32 : 1	68 : 1	100 : 1	15	79.27
9	32 : 1	102 : 1	50 : 1	5	38.66
*K*_1_	33.76	47.41	73.84	39.44	
*K*_2_	55.58	43.14	43.17	44.87	
*K*_3_	46.60	45.39	18.93	51.63	
*R*	21.82	4.27	54.91	12.19	

a A: the ratio of PC to Chol (n/n); B: the ratio of PC to DSPE-mPEG2000 (n/n); C: the ratio of PC to LPC (n/n); D: ultrasonic time, *K*_1_, *K*_2_ and *K*_3_ are the mean response values of corresponding levels. *R* represents the extreme difference of each factor.

### Characterization of LPC-LP

**Fig. 1:**
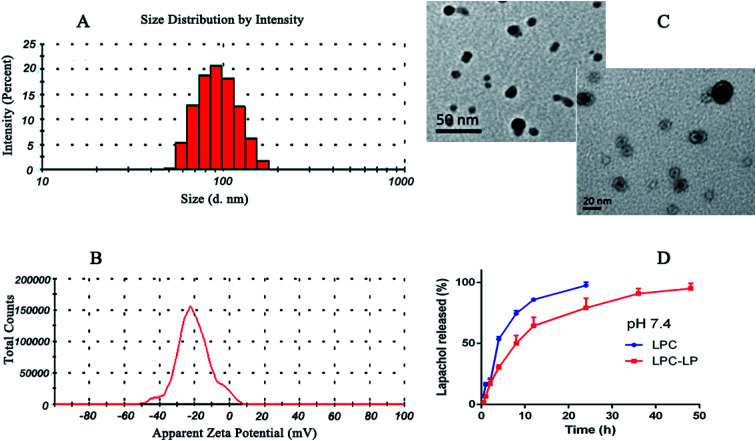
(A) Particle size distribution of LCP-LP; (B) zeta potential of LCP-LP; (C) TEM image of LCP-LP; (D) *in vitro* release profile of LPC and LPC-LP in pH 7.4 PBS.

The average particle size of the optimal formulation was 85.92 ± 2.35 nm with PDI of 0.30 ± 0.02, indicating LPC-LP had ideal particle size and narrow particle size distribution (Fig. 1A). The observation by transmission electron micrograph (TEM) showed that the liposomes were spherical and highly homogeneous in size (Fig. 1C), which could help to reduce the irritation of nanoparticles on blood vessels during the intravenous administration.^39^ The shape and size of the nanoparticles could directly affect the uptake of nanoparticles by cells, nanoparticles smaller than 100 nm were easier to take up by cells, and the sphere-shaped particles were significantly better than the rod-shaped particles.^52^ The ZP referred to the electric potential difference between the surface of nanoparticles and the solution. Generally, the larger the absolute value of the potential, the higher the charge repulsive force among the nanoparticles.^5^ Zeta potential above 30 mv was usually recommended to prevent particles from contacting and agglomerating. Such a dispersed system was relatively stable.^47^ The ZP of LPC-LP was −40.70 ± 9.20 mV (Fig. 1B), indicating that the liposome had excellent stability.

The *in vitro* release profile of lapachol from LPC-LP and LPC with equal doses was obtained by graphing the cumulative percentage of the drug released in the release medium as a function of the time. As shown in Fig. 1D, free lapachol was released rapidly from LPC in the first 4 h with the cumulative release amount up to 53.50%, and the release was almost finished at 24 h (97.51%). Whereas, the release rate of LPC-LP decreased significantly, comparing with that of LPC, indicating that release of lapachol from LPC-LP was delayed. The main reason was that the liposome acted as a controlled system, and its membrane was a kind of rate-limiting membrane. Therefore, it took time for encapsulated drugs to release by dissolution and diffusion from the liposomal membrane.^53^ Biphasic release behaviour of lapachol was observed from the LPC-LP with 30% rapid drug release within the initial 4 h, followed by a slow sustained release for 48 h. The reason for the initial rapid release was the release of lapachol adhered to the surface of nanoparticles. Whereas, the lapachol encapsulated in the core of LPC-LP released gradually.^38,54^ Although there was a burst release phenomenon in the initial stage of LPC-LP, the lapachol liposome could significantly prolong the release of lapachol from LPC-LP, indicating that LPC-LP had sustained and controlled-release ability. In order to investigate the mechanism of drug release, various release models were used to fit the *in vitro* release data of LPC-LP by DD Solver software, including first-order, Higuchi, zero-order and Weibull kinetic models. The best fit model was determined by correlation coefficients (*R*^2^).^45^ Finally, the Weibull model was found to be the optimal fit, with the equation of ln(*F* − 100) = −4.6 + *t*^0.8^/7.8 (*R*^2^ = 0.9688).

### Validation of analytical method

**Fig. 2:**
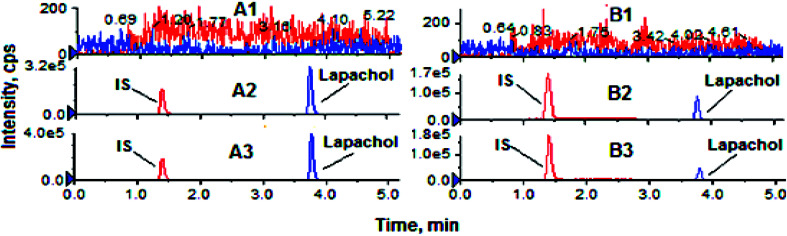
Representative UPLC-MS/MS chromatograms for lapachol and IS. (A1) Blank rat plasma; (A2) blank plasma spiked with lapachol (1 μg ml^−1^) and IS; (A3) rat plasma sample 12 h after administration of LPC-LP; (B1) blank mouse brain; (B2) blank mouse brain homogenate spiked with lapachol (200 ng ml^−1^) and IS; (B3) mouse brain homogenate 0.17 h after administration of LPC-LP.

The results for the selectivity and specificity were shown in Fig. 2, the retention times for IS and lapachol were approximately 1.4 and 3.7 min, respectively. No endogenous substances interfered with lapachol and IS were found in two biological matrices, suggesting that the method exhibited good selectivity. The calibration curves for the determination of lapachol in the plasma were divided into 5–1000 ng ml^−1^ (low concentration range) and 1–100 μg ml^−1^ (high concentration range) sections. For low concentration range, the regression equation of the calibration curve was *y* = 0.00055*x* + 0.00314 (*R*^2^ = 0.999), while for the high concentration range, the calibration curve had the equation of *y* = 0.1546*x* + 0.37771 (*R*^2^ = 0.998). In addition, the linear calibration curve for the brain tissue was also identified within the range of 5–1000 ng ml^−1^ (*y* = 0.00036*x* + 0.01462 *R*^2^ = 0.998). Therefore, the method proved to have excellent linearity. The LLOQ was 5 ng ml^−1^ for both biological matrices. Compared with assays for quantification of lapachol reported previously,^27^ the method we established had lower LLOQ, indicating that it was more sensitive. Furthermore, this method had wider detection concentration range and shorter detection time. The results of the precision and accuracy of the method for both kinds of biological samples were shown in Table 3, the intra-day and inter-day precision (RSD%) were all less than 12.09%, while the accuracy (RE%) ranged from −13.11% to 12.66%. The values of precision and accuracy were all within the acceptable limits, indicating that the method we developed was reliable. The results of recovery for lapachol were between 93.38% and 112.56%, and the data of the matrix effect was in the range of 87.4–112.52% for all the QC samples (Table 4). These results indicated that the recovery for lapachol was acceptable according to FDA guidances, and there was no significant matrix effect in the plasma and brain homogenate. In stability studies, the variation for all the QC samples was within ±14%, and the RSD% was less than 13% (Table 5), indicating that analytes were stable in both biological matrices under analysis conditions. In summary, the developed method for quantification of lapachol has been well validated according to FDA recommended guidelines. It is appropriate for analysis of lapachol *in vivo*.

**Table 3:** Accuracy and precision of the UPLC/MS method for lapachol in the plasma and brain (*n* = 4)

Matrix	Spiked	Intra-day			Inter-day		
		Measured mean ± SD	Precision (RSD%)	Accuracy (RE%)	Measured mean ± SD	Precision (RSD%)	Accuracy (RE%)
Plasma (ng ml^−1^)	5	4.96 ± 0.56	11.34	−0.77	5.04 ± 0.28	5.47	0.86
	100	105.10 ± 12.70	12.09	5.10	94.97 ± 5.48	5.77	−5.03
	1000	936.53 ± 41.71	4.45	−6.35	960.15 ± 42.04	4.38	3.99
Plasma (μg ml^−1^)	1	1.06 ± 0.12	11.00	6.40	0.96 ± 0.06	5.89	−3.66
	20	21.94 ± 1.52	8.42	9.71	21.33 ± 1.07	5.03	6.67
	100	86.90 ± 2.45	2.82	−13.11	94.07 ± 2.62	2.78	−5.93
Brain (ng ml^−1^)	5	5.58 ± 0.43	7.74	11.59	5.11 ± 0.22	4.24	2.29
	100	101.68 ± 9.67	9.51	1.68	98.45 ± 3.88	3.95	−1.55
	1000	940.05 ± 49.77	5.29	−6.00	1016.80 ± 11.83	1.16	1.68

**Table 4:** Recovery and matrix effect of lapachol in the plasma and brain (*n* = 4)

Matrix	Spiked	Recovery		Matrix effect	
		Mean ± SD (%)	RSD%	Mean ± SD (%)	RSD%
Plasma (ng ml^−1^)	5	109.74 ± 9.63	8.77	106.80 ± 17.53	16.43
	100	103.50 ± 11.05	10.68	106.45 ± 16.24	15.26
	1000	106.06 ± 9.12	8.60	87.40 ± 2.48	2.83
Plasma (μg ml^−1^)	1	106.19 ± 9.24	8.70	94.58 ± 13.26	14.02
	20	103,30 ± 11.43	11.07	100.66 ± 2.56	2.54
	100	112.56 ± 1.73	1.54	101.36 ± 6.89	6.06
Brain (ng ml^−1^)	5	110.74 ± 6.65	6.01	112.52 ± 8.38	7.45
	100	94.70 ± 2.64	2.78	110.36 ± 5.00	4.53
	1000	93.38 ± 4.72	5.06	109.75 ± 7.90	7.19

**Table 5:** Stability of lapachol in the plasma and brain under different storage conditions (*n* = 4)

Matrix	Spiked	Room temperature for 12 h			Storage for 24 h at 4 °C			Storage for 48 h at −20 °C		
		Measured mean ± SD	RSD (%)	RE (%)	Measured mean ± SD	RSD (%)	RE (%)	Measured mean ± SD	RSD (%)	RE (%)
Plasma (ng ml^−1^)	5	5.40 ± 0.42	7.81	8.01	5.37 ± 0.55	10.2	7.43	5.17 ± 0.67	12.88	3.39
	100	106.48 ± 4.64	4.36	6.48	87.31 ± 1.88	2.16	−12.69	105.08 ± 5.75	5.47	5.08
	1000	1088.33 ± 79.64	7.32	8.83	971.23 ± 40.16	4.13	−2.88	909.58 ± 40.63	4.47	−9.04
Plasma (μg ml^−1^)	1	0.92 ± 0.12	12.56	−7.84	0.92 ± 0.06	6.19	−7.98	0.88 ± 0.03	3.87	12.35
	20	22.51 ± 0.25	1.12	12.57	20.74 ± 0.61	2.93	3.69	22.64 ± 0.25	1.09	13.19
	100	87.80 ± 3.87	4.41	−12.20	88.65 ± 1.82	2.05	−11.36	94.71 ± 1.55	1.64	−5.29
Brain (ng ml^−1^)	5	5.63 ± 0.32	5.75	12.52	4.92 ± 0.75	15.22	−1.7	5.39 ± 0.35	6.5	7.72
	100	89.43 ± 3.59	4.02	−10.58	92.59 ± 2.98	3.22	−7.41	103.64 ± 8.46	8.17	3.63
	1000	1063.18 ± 45.18	4.25	6.32	964.45 ± 81.65	8.47	−3.56	966.98 ± 55.68	5.76	−3.3

### Pharmacokinetics and brain distribution of formulations

**Fig. 3:**
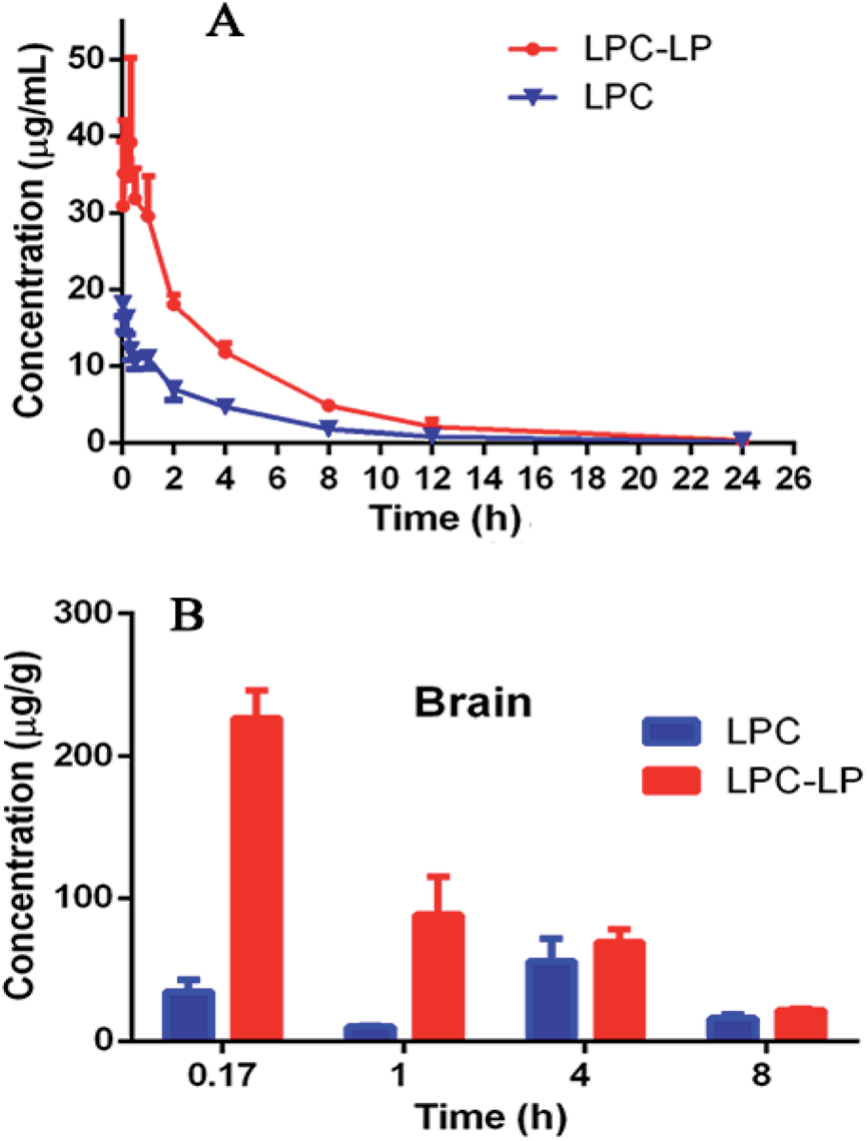
(A) Pharmacokinetic profile of LPC and LPC-LP in rats (*n* = 6); (B) brain distribution of LPC and LPC-LP at 0.17, 1, 4, and 8 h, respectively (*n* = 4).

The plasma concentration–time profile of lapachol from two formulations in rats after single i.v. administration of 6.8 mg kg^−1^ was shown in Fig. 3A. LPC displayed a low level of lapachol in plasma, and could not be detected at 12 h after administration. When lapachol was loaded into liposome nanoparticles, the concentration of lapachol in plasma was much higher and could be quantified in plasma for longer time than that of LPC. Table 6 summarized the pharmacokinetic parameters of LPC and LPC-LP. The results showed that LPC-LP prolonged the elimination half-life (*t*_1/2β_) of lapachol in plasma by 2.4 times, compared with LPC. The clearance rate of LPC and LPC-LP was 0.14 l h^−1^ kg^−1^ and 0.04 l h^−1^ kg^−1^ respectively, which indicated the elimination rate of LPC-LP in rat was 3.5 times slower than that of LPC. The AUC of LPC-LP was greater than that of LPC. The relative bioavailability of LPC-LP was 310.73%. In summary, LPC-LP effectively prolonged the systemic circulation time, increased the plasma exposure of lapachol, thereby, improved the efficacy of lapachol.

**Table 6:** Pharmacokinetic parameters of lapachol after single i.v. administration of LPC and LPC-LP (6.8 mg kg^−1^) to rats (*n* = 6)^a^

Parameters	Unit	LPC	LPC-LP
*T*_1/2α_	Hour	0.38 ± 0.76	2.06 ± 1.87
*T*_1/2β_	Hour	2.42 ± 0.70	5.81 ± 1.75**
AUC (0 − *t*)	(μg ml^−1^) h	52.65 ± 20.10	161.71 ± 29.98**
AUC (0 − ∞)	(μg ml^−1^) h	53.51 ± 20.23	166.27 ± 31.44**
CL	mg kg^−1^ h^−1^ (μg ml^−1^)^−1^	0.14 ± 0.06	0.04 ± 0.01**
*C*_max_	μg ml^−1^	20.45 ± 4.33	47.82 ± 24.48*

a *P < 0.05 and **P < 0.01 *vs.* the LPC group.

The Fig. 3B showed the brain distribution of lapachol from LPC and LPC-LP. As expected, the concentration of LPC-LP was higher than that of LPC at each time point. The *C*_max_ and AUC of LPC-LP were 226.5 ± 38.1 ng g^−1^ and 617.8 ± 91.4 (ng g^−1^) h. Whereas, the *C*_max_ and AUC of LPC were 55.5 ± 32.1 ng g^−1^ and 260.2 ± 107.1 (ng g^−1^) h. Compared with LPC, the *C*_max_ and AUC of LPC-LP in brain significantly increased by 4.1 and 2.4 times respectively, suggesting that LPC-LP significantly altered the brain exposure of lapachol, and increased its accumulation in the brain. This result was in good agreement with previously reported results, which showed that liposome surface-modified PEG appeared to be a promising brain drug delivery.^55^

In our study, LPC-LP altered the trajectory of lapachol in the body by prolonging the half-life *in vivo*, reducing the clearance rate, and increasing brain intake. In the treatment of brain diseases, that was what we expected. Liposome could reduce dosage of drugs, prolong the dosing interval, and increase the efficacy.^47^ The long-term circulation and brain targeting of LPC-LP might be related to a variety of factors. Firstly, the liposome was a sustained-release system which released lapachol slowly and continuously in the body. And liposome could protect unreleased lapachol from degradation by enzymes and acids *in vivo*;^38,51^ moreover, materials for liposomes were also very important factors. The surface modification of liposomes with PEG could avoid recognition and phagocytosis by the reticuloendothelial system (RES), improve the stability of the liposome, prolong the retention time of drugs effectively, increase the drug concentration *in vivo*, and thereby improve the drug penetration through BBB.^7,55–58^ In this study, lecithin was used as a surfactant of liposome. Lecithin was a significant component of the cell membrane, so liposomes could rapidly fuse with cell membranes to enter target cells;^37^ in addition, previous studies showed that the smaller the particle size of the drug, the easier it was to pass through the BBB. The lipid nanoparticles with particle size less than 150 nm were more potent through BBB.^59^ The particle size of LPC-LP prepared in this study was below 100 nm, further indicating that it had good brain targeting; besides, PEG liposome itself might also affect some critical physiological pathways on BBB, such as efflux transporters.^55^ It could reduce the efflux of drugs, increase the accumulation of drugs in the brain, and then improve the efficacy.

### Cytotoxic effects of LPC-LP and LPC on C6 cells

**Fig. 4:**
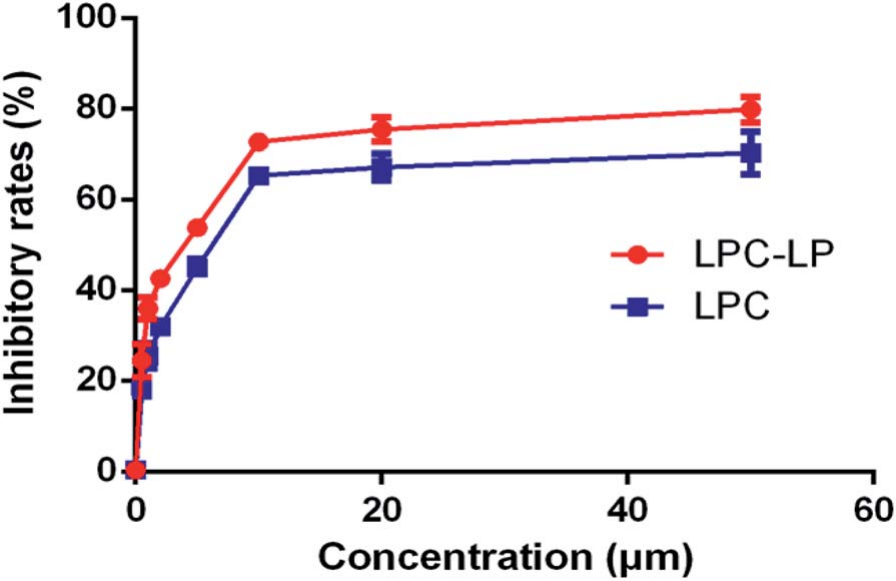
The inhibitory rate of LPC-LP and LPC on C6 cell (*n* = 3).

As shown in Fig. 4, both LPC and LPC-LP could inhibit the proliferation of C6 cells in a dose-dependent manner, and IR of LPC-LP on C6 cells was higher than that of LPC at different concentrations (*P* < 0.01). The IC_50_ values of LPC-LP and LPC on C6 cells were 2.69 μM and 4.90 μM respectively, which meant that the inhibition of C6 cell proliferation was enhanced when lapachol was encapsulated in liposomes. The results might be attributed to the physicochemical properties of LPC-LP with high lipophilicity and excellent biocompatibility. Compared with LPC, LPC-LP was easier to fuse with the cell membrane and be taken up by cells, thereby improve the therapeutic effect.^5^ It should be emphasized that this result should be further verified by *in vivo* experiments.

## Conclusions

In the present study, LPC-LP was successfully optimized and prepared to overcome the defects of lapachol with poor water solubility, short half-life and low brain distribution. The characterizations of the optimized formulations were evaluated, including the particle size, ZP, EE, and release profile. Meanwhile, a sensitive and rapid UPLC-MS/MS method was developed and validated for the determination of lapachol in two biological matrices. It was successfully applied to the study on the pharmacokinetics and brain distribution of LPC-LP for the first time. LPC-LP could regulate physicochemical properties of lapachol, improve its pharmacokinetic profile and increase brain intake. The cytotoxicity study *in vitro* further confirmed that LPC-LP enhanced the inhibition of C6 cells and increased the anti-glioma effect. In summary, LPC-LP was a promising and potential nanoparticle in the treatment of glioma.

## Conflicts of interest

There are no conflicts to declare.

## Supplementary Material
